# Extraction of Important Factors in a High-Dimensional Data Space: An Application for High-Growth Firms

**DOI:** 10.3390/e25030488

**Published:** 2023-03-10

**Authors:** Takuya Wada, Hideki Takayasu, Misako Takayasu

**Affiliations:** 1 Department of Mathematical and Computing Science, School of Computing, Tokyo Institute of Technology, Yokohama 226-8502, Japan; 2 Institute of Innovative Research, Tokyo Institute of Technology, Yokohama 226-8502, Japan; takayasu@csl.sony.co.jp; 3 Sony Computer Science Laboratories, Tokyo 141-0022, Japan

**Keywords:** variable selection, feature selection, high-growth firms, Bayesian method, big data

## Abstract

We introduce a new non-black-box method of extracting multiple areas in a high-dimensional big data space where data points that satisfy specific conditions are highly concentrated. First, we extract one-dimensional areas where the data that satisfy specific conditions are mostly gathered by using the Bayesian method. Second, we construct higher-dimensional areas where the densities of focused data points are higher than the simple combination of the results for one dimension, and then we verify the results through data validation. Third, we apply this method to estimate the set of significant factors shared in successful firms with growth rates in sales at the top 1% level using 156-dimensional data of corporate financial reports for 12 years containing about 320,000 firms. We also categorize high-growth firms into 15 groups of different sets of factors.

## 1. Introduction

We consider the general problem of extracting areas in a high-dimensional data space where points that satisfy specific conditions are concentrated. Generally, as factors associated with a specific condition are often unknown, we use the most available factors and examine their relevance to a particular condition [1]. However, the majority of the factors used are irrelevant or redundant, resulting in problems such as reduced accuracy of the analysis and increased analysis time [1,2]. Therefore, we are reducing the number of variables, a process called variable selection. Variable selection has various advantages, such as accuracy increase, analysis time reduction, and overfitting avoidance [2,3,4]. Many models have been proposed for this variable selection and used in various fields [4,5,6]. In recent years, machine learning models have been used to improve the accuracy of variable selection. For example, Genuer used random forests [7] to select significant variables in high-dimensional classification problems [8]. Grandvalet proposed a model that automatically performs relevance judgments and feature selection on support vector machines [9] and showed its effectiveness in facial expression recognition tasks [10]. However, machine learning models also have disadvantages; for example, generally their results are difficult to understand logically due to the complexity of these models and their black-box structure [11,12]. In addition, to the best of our knowledge, a general method for exhaustively extracting areas where the data that satisfy specific conditions are highly concentrated has not been established in the study of big data.

In this paper, we propose a new method based on a non-black-box model to solve this general problem. We use indicators calculated using the Bayesian method and Szymkiewicz-Simpson coefficient as evaluation measures for variable selection and extraction of pairs of variables, respectively. The Bayesian method is a data analysis method that uses existing information [13,14]. This point differs from the likelihood method and gives the advantage of more flexible model assumptions and facilitating statistical inference even for complex problems [15,16]. We use the Bayesian method, which is used in various fields, including ecology and seismology [14,15,17,18,19], to construct the posterior distribution of a specific indicator. Then we use the lower limit of the confidence interval as a new indicator for the evaluation measure. As a basic tool of data analysis, we introduce the Szymkiewicz–Simpson coefficient, which quantitatively evaluates the degree of overlap between two sets [20].

In this study, we analyze the factors that contribute to a firm’s high growth as an example of the application of this model. Firm growth is significant and attracts the attention of investors and banks [21,22]. Demirgüç-Kunt clarified that a firm’s growth is related to the financial and legal system [23]. Baum extracted venture growth factors with structural equation modeling and data on 17 predictor variables [24]. We analyze the factors of a firm’s growth using machine learning models in recent years. Van Witteloostuijn and Kolkman analyzed the factors that contribute to a firm’s growth using random forests [25]. Among them, the phenomenon of high growth is heterogeneous, and Delmar showed that it can be classified into seven groups via cluster analysis [26]. Coad forecasted high-growth firms with Lasso [27], a machine learning model [28]. We identify high-growth patterns using our model and verify them with Delmar’s and Coad’s results.

The remainder of this paper is organized as follows. Section 2 explains the dataset and defines each firm’s growth rate in sales and high-growth firms. Section 3 describes the mathematical basis used in this methodology and methods. We first determine the posterior distribution of the probability that a firm will grow high within a particular area using the Bayesian method and then define the existence probability of high-growth firms. We also provide proof of the formulas used in Section 4 and the subsequent sections. Section 4 describes step-by-step the results of the method and classifies the high-growth firms into 15 groups based on different factors. In Section 5, we discuss the advantages, considerations, concerns, comparison with previous studies, and indicators of analysis. Finally, Section 6 describes our results and the potential applications of our method.

## 2. Data

In this study, we use the corporate financial dataset provided by TEIKOKU DATABANK, Ltd. (TDB). In Japan, companies often ask a third-party corporate credit research organization to obtain information about a firm when they are looking for new business partners to expand sales or to check the business condition of existing business partners. TDB is one of the largest corporate credit research providers in Japan and has been providing corporate credit research for more than a century [29]. In this study, we use 12 years of data from 2005 to 2016 with sales data existing for the next three years contained in this corporate financial dataset. The data include about 320,000 firms with 1.7 million data points. The first 10 years of the 12 years of data are used for the analysis, and the remaining 2 years are used for validation. Note that the dataset is not complete, and some financial items are missing in some firms. In such cases, we simply neglect missing items in our analysis. As a result, the number of firms in each financial item becomes equal to the total number of firms minus the number of missing data for the item.

We focus on the rate of increase in sales for each firm, which is defined by the following equation:

(1) $$\mathrm{Growth}\;\mathrm{rate}\;\mathrm{in}\;\mathrm{sales}=\frac{\mathrm{Current}\;\mathrm{sales}\;\mathrm{after}\;3\;\mathrm{years}}{\mathrm{Current}\;\mathrm{sales}}$$

In this paper, we define high-growth firms as ones whose growth rate is in the top 1% of all firms in each analysis or verification data. Specifically, a high-growth firm has a growth rate of 4.913 times or higher for the analysis data and 4.428 times or higher for the validation data. We use our method to extract the conditions commonly satisfied by these high-growth firms in financial items. We exclude financial items that have a very strong correlation (correlation coefficient of higher than 0.95) with the current sales used in the definition of growth rate in sales to avoid false correlations. We consider 156 financial items, such as the capital and current ratio in general.

To verify whether high-growth firms are dense not by coincidence, we randomly shuffle the 10 years of data from 2005 to 2014 for comparison. Namely, we create five sets of randomly shuffled data by using the command “shuffle” in Python for each of the 156 financial items with pseudorandom numbers generated by PCG64 [30].

We apply our method explained in the following Section 3 to the 10 years of real data and the five sets of randomly shuffled data.

## 3. Method

In this section, we explain the definition of the existence probability of high-growth firms used in the analysis and show how to calculate the existence probability of high-growth firms when the conditions are independent (in Section 3.1). We describe the analytical procedure of our method (in Section 3.2).

### 3.1. Mathematical Basis

Let *q* be the existence probability of high-growth firms in a specific area *J*, *a* be the number of high-growth firms, and *b* be the number of non-high-growth firms. The probability of occurrence conditioned by *q*, f(a,b|q), fulfills the following equation:

(2) f(a,b|q)=a+baqa(1−q)b

Then, using Bayesian analysis with the prior distribution π(q), the posterior distribution π(q|a,b) of *q* conditioned by *a* and *b* is given as follows:

(3) $$\pi \left(q|a,b\right)=\frac{\pi (q)f(a,b|q)}{\int_{0}^{1}\pi \left(q\right)f\left(a,b|q\right)dq}$$

Here, we use the conjugate prior $\pi \left(q\right)\propto q^{\alpha }{\left(1-q\right)}^{\beta }$, which is a beta distribution with parameters α+1 and β+1, for the prior distribution of binomial distribution to reduce computational effort during the analysis. In addition, we condition that E[q|a=0,b=0]=r and α+β=0; that is the expectation of probability *q* in the case of no sample data is equal to r=0.01. Then, α=−β=2r−1, and π(q|a,b) is obtained as follows:

(4) $$\pi \left(q|a,b\right)=\frac{\Gamma (a+b+2)}{\Gamma (a+2r)\Gamma (b-2r+2)}q^{a+2r-1}{\left(1-q\right)}^{b-2r+1}$$

From this posterior distribution, we estimate the lower bound of the probability of the existence of high-growth firms with a 99% confidence interval. That is, we regard the existence probability in the area *J* with *a* and *b* by the value of *y*, which is determined by solving the following equation, the inverse of the regularized incomplete beta function.

(5) $$r=\frac{\Gamma (a+b+2)}{\Gamma (a+2r)\Gamma (b-2r+2)}\int_{0}^{y}q^{a+2r-1}{\left(1-q\right)}^{b-2r+1}dq$$

We apply this 99% confidence value for the extraction of one to multi-dimensional areas.

Here, we prove the basic equation, which is used in the following sections for the extraction of two- or higher-dimensional areas. We consider particular conditions 1 to *n* and let $A_{1}$ to $A_{n}$ be flag variables that specify these conditions. For example, $A_{n}=1$ implies that condition *n* is fulfilled. In addition, let *X* be a flag variable that indicates whether high growth has occurred. We assume that $A_{1}$ to $A_{n}$ are independent of each other and also independent under the condition of X=0, namely, for non-high growth cases. The probabilities of satisfying the conditions from 1 to *n*, $P(A_{1}=1,A_{2}=1,\ldots ,A_{n}=1)$, and from 1 to *n* under X=0, $P(A_{1}=1,A_{2}=1,\ldots ,A_{n}=1|X=0)$, are given as follows:

(6) $$P\left(A_{1}=1,A_{2}=1,\ldots ,A_{n}=1\right)=\prod_{i=1}^{n}P\left(A_{i}=1\right)$$

(7) $$P\left(A_{1}=1,A_{2}=1,\ldots ,A_{n}=1|X=0\right)=\prod_{i=1}^{n}P\left(A_{i}=1|X=0\right)$$

Then, under the conditions from 1 to *n* fulfilled, the existence probability of high-growth firms $P(X=1|A_{1}=1,A_{2}=1,\ldots ,A_{n}=1)$ can be calculated using these equations with Bayes’ formula as follows:

(8) $$P\left(X=1|A_{1}=1,A_{2}=1,\ldots ,A_{n}=1\right)=1-\frac{\prod_{i=1}^{n}1-P\left(X=1|A_{i}=1\right)}{{\left(1-P\left(X=1\right)\right)}^{n-1}}$$

### 3.2. Method

**Figure 1:**
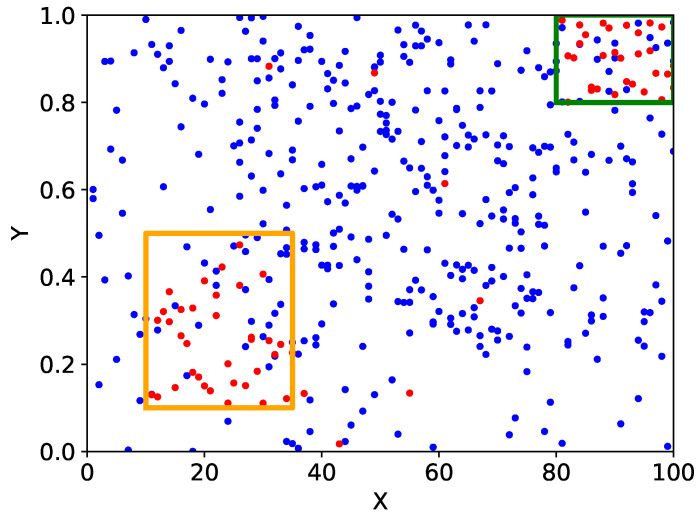
Schematic of our method if it were two-dimensional. The red dots represent high-growth firms, the blue dots represent non-high-growth firms, and the orange and green boxes are the areas to be extracted as high density areas.

We consider the financial data as a distribution of points in a 156-dimensional space with 156 financial items as variables and then search for areas with high concentrations of points of high-growth firms. Figure 1 shows an image of this model if it were two-dimensional.

Our analysis involves five steps:

Step1 Extraction of one-dimensional areas for each financial item;
Step2 Reduction of areas containing similar data points;
Step3 Extraction of two-dimensional areas;
Step4 Extraction of higher-dimensional areas;
Step5 Grouping.

#### 3.2.1. Step1. Extraction of One-Dimensional Areas for Each Financial Item

**Figure 2:**
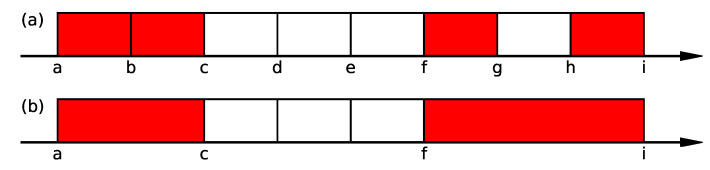
Schematic of Step1. (**a**) We divide the axis into non-overlapping segmented areas where at least 5% of the data points are included. For each area, we calculate the existence probability using Equation (5), and if it is higher than 0.01, the area is colored in red. In this case, four areas are extracted: [a,b), [b,c), [f,g), and [h,i). (**b**) We merge neighboring areas into one area as shown for [a,c), and [f,i) if the merged area’s existence probability is higher than 0.01.

In Step1, we extract high-concentration areas of high-growth firms in one dimension. A schematic of this step is presented in Figure 2. First, we project the points in a 156-dimensional space onto a single coordinate axis. Second, we segment the data into non-overlapping intervals, including at least 5% of the data. Third, in each separated area, we calculate the existence probability of high-growth firms using Equation (5) with the numbers of high-growing and non-high-growth firms. Subsequently, we extract areas where the existence probability is higher than 0.01 with the 99% confidence. It should be noted that the proportion of high-growth firms in each financial item depends on the number of missing data, and there are items whose whole proportions of high-growth firms exceed 0.01. For such financial items, we set the threshold values of extraction by the value of the whole proportion for each item instead of 0.01. The schematic of the procedure up to this point is presented in Figure 2a. In this case, four areas are extracted: [a,b), [b,c), [f,g), and [h,i).

When multiple areas are extracted in one dimension, we check the possibility of combining the areas. The schematic of this procedure is presented in Figure 2b. For the extracted areas that are adjacent to each other, they are combined, as schematically shown by the interval [a,c) in Figure 2b. If there is an unselected area in between, as shown by the interval [g,h) in Figure 2a, the existence probability of high-growth firms in the connected area is calculated by using Equation (5). If it exceeds 0.01, these areas are merged, as shown by the interval [f,i) in Figure 2b. This process is continued until no more areas can be combined.

#### 3.2.2. Step2. Reduction of Areas Containing Similar Data Points

In Step2, we reduce overlapping areas, which are extracted in Step1 based on the similarities defined below. Let us denote the set of firms in area *A* extracted from financial item $\tilde{a}$ as $\tilde{A}$. Note that the whole space is 156-dimensional, and this area is defined by the restricted range only for item $\tilde{a}$; thus, all other items can take any value in this set. If another item $\tilde{b}$ is similar to item $\tilde{a}$, then firms $\tilde{B}$ in the extracted area *B* may overlap with $\tilde{A}$. For a quantitative evaluation of such overlap, we introduce the Szymkiewicz–Simpson coefficient defined as follows:

(9) $$Szymkiewicz-Simpson\;coefficient=\frac{|\tilde{A}\cap \tilde{B}|}{min(|\tilde{A}|,|\tilde{B}|)}$$

We calculate this indicator for all combinations of two areas chosen from the areas extracted in Step1 and observe the cumulative distribution function of this indicator. From the shape of the distribution, we introduce a threshold value of this indicator and delete these areas with higher indicators than the threshold. Detailed processes are discussed in Section 4.2.

#### 3.2.3. Step3. Extraction of Two-Dimensional Areas

In Step3, we extract the two-dimensional areas where the existence probability of high-growth firms is higher. Subsequently, we calculate the existence probability of high-growth firms for all two-dimensional areas characterized by the direct product of the two conditions chosen from the areas after Step2. When the probability of a two-dimensional area estimated by using Equation (5) is less than that calculated using Equation (8), which assumes the independence of two financial items, then the two-dimensional area is aborted.

#### 3.2.4. Step4. Extraction of Higher-Dimensional Areas

In Step4, we extract high-dimensional areas where the existence probability of high-growth firms is higher than the value of independent direct products estimated using Equation (8). For the two-dimensional area chosen in Step3 with the highest existence probability of high-growth firms, we add another one-dimensional condition that is chosen from Step2 and not already used in two-dimensional conditions. For all conditions in Step2, we calculate the existence probabilities of the combined three-dimensional areas using Equation (5) and choose the case that provides the highest existence probability of high-growth firms. If this probability is higher than the value estimated using Equation (8) and the existence probability of high-growth firms before adding the condition, then we assume that the new three-dimensional area’s density of high-growth firms is significantly higher than the case of independent direct products. Thus, we adopt this three-dimensional area. If this condition is not fulfilled, then the two-dimensional area condition is kept two-dimensional. We proceed to the process for the 2nd candidate of the two-dimensional area chosen in Step3 and repeat the same procedure, followed by the 3rd and 4th, etc., to all two-dimensional candidates. For the newly adopted three-dimensional area, we add another one-dimensional condition chosen from Step2 as before and construct four-dimensional areas. We find the case that provides the highest existence probability of high-growth firms. Similarly, if the probability estimated using Equation (5) is higher than the value of Equation (8), we adopt the four-dimensional area. These processes of finding higher-dimensional areas are completed if there remains no combination of a higher-dimensional area that satisfies a certain condition; that is the probability of high-growth firms estimated using Equation (5) exceeds the value of Equation (8), and the existence probability of high-growth firms is higher than before the condition is added.

For the areas obtained in these processes, we verify whether the existence probability of high-growth firms is also increased in the data for validation. The procedure is used to add conditions in the same order as the conditions for the areas obtained in these processes until the existence probability of high-growth firms stops increasing. Using this procedure, we examine the validity of the obtained higher-dimensional areas and select high-dimensional areas that are non-local and have a high existence probability of high-growth firms. For the selected areas, the following process is followed to determine the areas of focus:

1. Remove high-dimensional areas that have the same set of conditions.
2. Remove similar high-dimensional areas where all firms in the area match, despite not being under the same conditions.
3. If the inclusion relationship is established, remove the area with the smallest number of firms.

#### 3.2.5. Step5. Grouping

In Step5, we define groups of the high-dimensional areas selected in Step4 using hierarchical clustering using the Ward method [31] with the measure of the dissimilarity between areas given as follows:

(10) $$dissimilarity=1-\frac{|\overset{ˋ}{A}\cap \overset{ˋ}{B}|}{min(|\overset{ˋ}{A}|,|\overset{ˋ}{B}|)}$$

where $\overset{ˋ}{A}$ and $\overset{ˋ}{B}$ are groups of high-growth firms belonging to areas *A* and *B*, respectively. We set the dissimilarity threshold to a value where most high-dimensional areas in the same group contain the same condition. The detailed process is discussed in Section 4.5.

## 4. Results

We define the abbreviated names for commonly used financial items, conditions, and units in Table 1.

### 4.1. Extraction of One-Dimensional Areas for Each Financial Item

Step1 extracted 197 areas of 143 financial items. The top five areas with the highest existence probability of high-growth firms are presented in Table 2.

**Figure 3:**
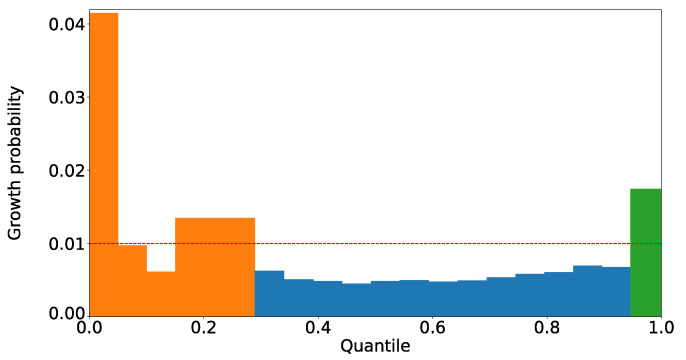
Existence probability of high-growth firms in each of the segmented areas, projected on the axis of the ratio of net income to sales (before amortization and after tax, %). The horizontal axis is the quantile from the beginning to the end of the segmented area, and the vertical axis is the existence probability of high-growth firms within the segmented area. The red dashed line represents 0.01, the percentage of high-growth firms in the overall area. For this financial item, the orange and green areas were extracted as the areas with densely populated high-growth firms, and the blue area was not extracted because it was not densely populated with high-growth firms. For the orange area, two areas were initially extracted: the 0–5.0% and 15.0–28.9% areas. These two areas and the areas in between where the existence probability of high-growth firms is low were merged into one area, as shown in Figure 2b.

The areas with the first and second highest existence probability of high-growth firms have a value of about 0.042. This implies that they are more than four times more densely populated with high-growth firms than normal ones. Two areas were extracted for each of the 54 financial items. The distribution of the existence probability of high-growth firms and details of the areas extracted for one example of those financial items are presented in Figure 3 and Table 3.

These orange and green areas are where high-growth firms are about 1.7 times more dense than normal ones. These areas are the two edges of the financial items, and it is thought that firms grow high due to different factors.

For validation, we performed the same one-dimensional extraction on five random data. We extracted 11, 11, 12, 13, and 13 areas, respectively. No multiple areas were extracted within a single financial item. The area with the highest existence of high-growth firms in these areas was about 1.08 times more dense than normal ones. These areas are used in Step2.

### 4.2. Reduction of Areas Containing Similar Data Points

**Figure 4:**
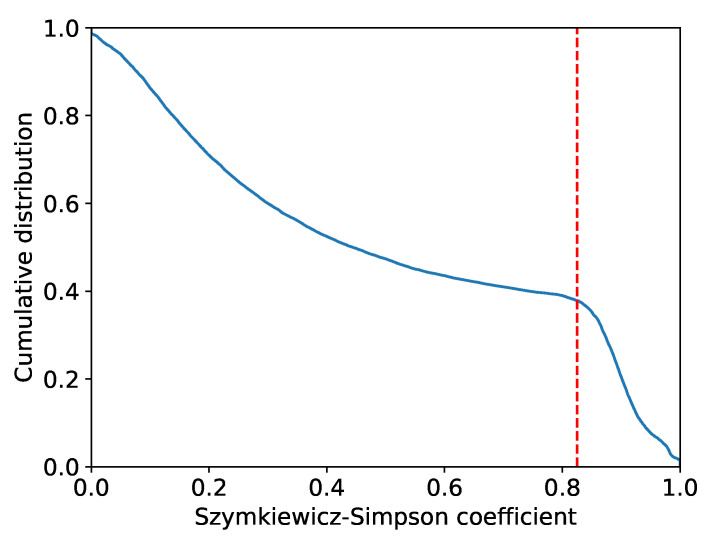
Cumulative distribution function of the values calculated for all combinations by using Equation (9). The horizontal axis is the value of the Szymkiewicz–Simpson coefficient, and the vertical axis is the cumulative distribution. The red dashed line represents 0.825, where the shape of the cumulative distribution function changes. This value was used as the threshold value.

Similar areas were deleted in Step2 for the 197 areas of 143 financial items extracted in Step1. The result of calculating Equation (9) for all combinations of the 197 areas is presented in Figure 4.

Figure 4 shows that the cumulative distribution function changes its slope around when the value of the Szymkiewicz–Simpson coefficient is 0.825. This value was used as the threshold value. In the combination of areas where the value of the Szymkiewicz–Simpson coefficient is greater than this value, the area with the smallest existence probability of high-growth firms was deleted. For example, the combination of an area with a turnover of current debt (months) of 7.44 or higher and an area with an increase/decrease in an investment of less than 0 (thousands of yen) resulted in a Szymkiewicz–Simpson coefficient value of 0.916. Therefore, we compared the existence probability of high-growth firms and removed the area with an investment volume of less than 0 (thousands of yen), which was a lower area. We finally extracted 67 areas of 51 financial items.

For the five random data, the highest Szymkiewicz–Simpson coefficient was about 0.24 in the combination obtained from the areas of financial items obtained in each. Considering that this is smaller than the threshold value of 0.825 in the data for analysis and that no similarity exists among the financial items and among the areas as the data were randomly shuffled, none of the areas were removed. The 11, 11, 12, 13, and 13 areas obtained in Step1 were used in Step3–Step5.

### 4.3. Extraction of Two-Dimensional Areas

The 67 areas of 51 financial items extracted in Step2 were used to extract the two-dimensional areas. We checked all possible combinations, and the top five two-dimensional areas with the highest existence probability of high-growth firms are presented in Table 4.

**Figure 5:**
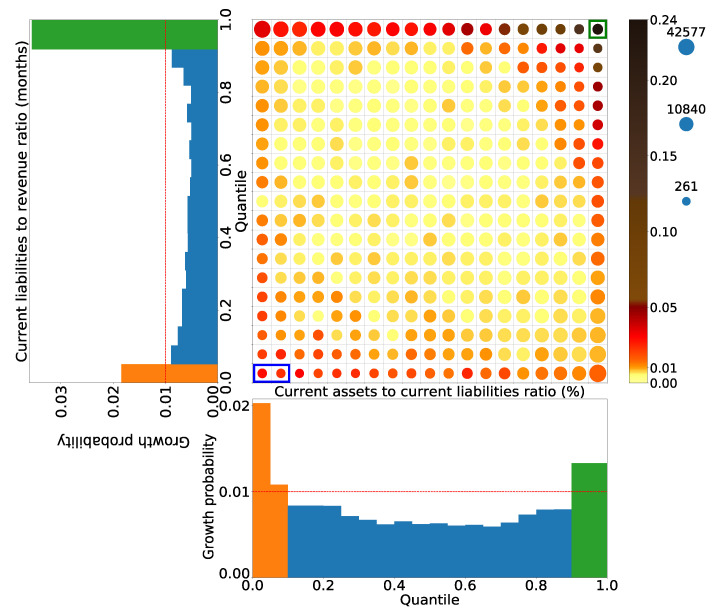
Extracted two-dimensional area of the first rank. The vertical and horizontal axes are divided by the current liabilities to revenue ratio (months) and the current assets to current liabilities ratio (%), respectively. The size of the circle represents the number of firms in the area, and the radius is scaled in a logarithmic scale. The colors of the circles represent the proportion of high-growth firms in the area. It is drawn in the order of yellow, orange, red, brown, and black, starting from the lowest to the highest. The green box at the right top is the area extracted as the two-dimensional area with the highest concentration of high-growth firms. The blue box at the left bottom is the area that was not extracted because the existence probability of high-growth firms in this area is lower than that of high-growth firms using Equation (8) if the two conditions are independent.

In the two-dimensional area where the existence of high-growth firms is in the first and second places, high-growth firms are about 20 times more dense than normal ones. Table 4 displays how many times the existence of high-growth firms is compared to when the two conditions are independent (Column Ratio), and these five areas are about five times as high. Therefore, some synergy must exist in the combination of these conditions. Figure 5 presents the extracted two-dimensional area of the first rank.

The green box area at the right top in Figure 5 is the area that satisfies the green areas in the turnover of current debt and the current ratio in the one-dimensional axes. It is 20 times more densely populated with high-growth firms than normal ones. It was also extracted as a two-dimensional area with the highest existence probability of high-growth firms. Meanwhile, the blue box area in Figure 5 is the area that satisfies the orange areas in the turnover of the current debt and the current ratio in the one-dimensional axes. The existence probability of high-growth firms in this area is 0.014. This value is lower than that of high-growth firms when the two conditions are independent, as calculated using Equation (8). Therefore, this area was not extracted as a two-dimensional area.

We obtain 2211 two-dimensional areas using the 67 conditions used for the 67 areas extracted in Step2. Among them, we extracted 1036 areas that are more densely concentrated with high-growth firms than that when the conditions were independent.

For the five random data, we check whether high-growth firms are densely populated in the two-dimensional areas using the conditions used for the areas extracted in Step2. The number of areas extracted as areas where the existence probability of high-growth firms is higher than that of high-growth firms calculated using Equation (8), under the condition that the two conditions are independent were 3, 4, 6, 7, and 9. Even in the area with the highest concentration of high-growing firms in any of the random data, the concentration of highest-growing firms is about 1.7 times the normal concentration. It was also about 1.5 times higher than when all conditions were independent, indicating no strong synergistic effect. These two-dimensional areas extracted as densely populated with high-growth firms in the random data are used in the analysis in step 4.

### 4.4. Extraction of Higher-Dimensional Areas

For the 1036 two-dimensional areas extracted in Step3, we extract 1036 high-dimensional areas by repeatedly adding the 67 conditions used in the 67 areas extracted in Step2. The top two high-dimensional areas that are extracted are presented in Table 5 and Table 6.

**Figure 6:**
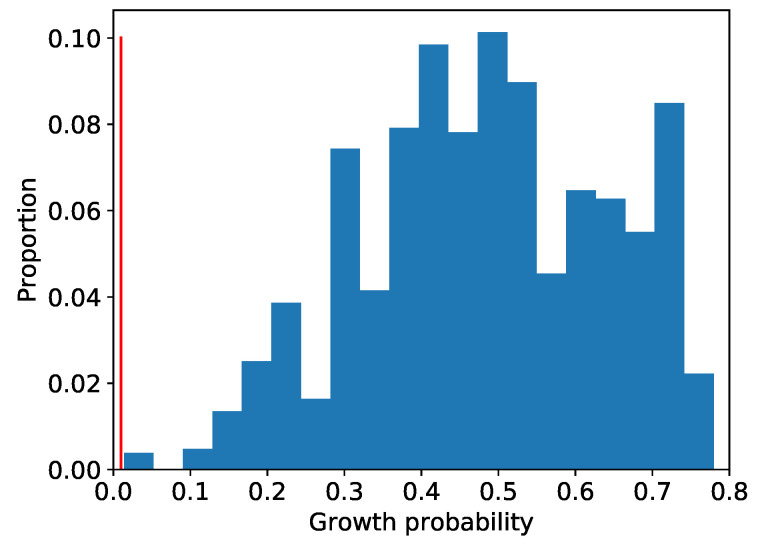
Distribution of the existence probability of high-growth firms in the high-dimensional areas. The vertical axis and horizontal axes are the proportion of 1036 areas and the existence probability of high-growth firms, respectively. The red line represents 0.01, the percentage of high-growth firms in the overall area.

The existence probability of high-growth firms decreased when the 8th and 9th conditions were added to the areas in Table 5 and Table 6. Therefore, the areas with the 7th and 8th dimensions in Table 5 and Table 6 were extracted as areas with a high concentration of high-growth firms. The existence probability of high-growth firms in these high-dimensional areas is about 0.77. This implies that high-growth firms in these areas are about 77 times more dense than normal ones. They are also about 5–7 times higher than that when all conditions were independent. Therefore, we can assume that some synergistic effects occur in the combinations of these conditions. As shown in Table 5 and Table 6, we extract the high-dimensional areas from the 1036 two-dimensional areas obtained in Step3. The distribution of the existence probability of high-growth firms in the high-dimensional areas finally obtained is presented in Figure 6.

As shown in Figure 6, 90% of the 1036 high-dimensional areas were able to extract areas where the high-growth firms are dense at 30 times or higher than the normal density. We have also extracted four areas where the high-growth firms are dense at less than three times the normal density, and all of these areas were two-dimensional ones. Subsequently, areas with a small number of data are called local ones. These areas became localized at the two-dimensional level, and no further high-dimensional areas could be extracted. Our method searched the entire area exhaustively, and the extracted areas include the local ones.

For the 1036 high-dimensional areas obtained in these processes, we verified whether the existence probability of high-growth firms is also increased in the data for validation. The verification procedure is to add conditions in the same order as the conditions for the areas obtained in these processes until the existence probability of high-growth firms stops to increase. As specific examples, the results of the verification in the areas of Table 5 and Table 6 are presented in the Table 7 and Table 8, respectively.

**Figure 7:**
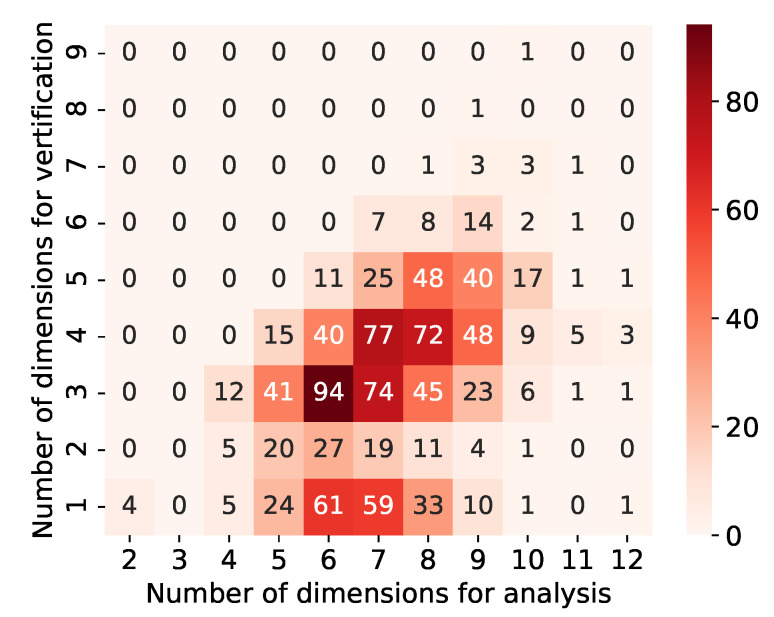
Distribution of the number of dimensions for which the existence probability of high-growth firms was maximized in the data for analysis and verification. The vertical and horizontal axes are the number of dimensions in verification data and analysis data, respectively. The numbers represent the number of areas with each dimension in the analysis and validation data. The colors indicate that the darker the red color, the higher the value, i.e., the greater the number of areas.

In the validation for both areas, the existence probability of high-growth firms decreased when the 5th condition was added. Thus, we confirmed the robustness of the results up to the four-dimensional area in these areas. In this validation, the existence probability of high-growth firms in the one-dimensional area in both validation results was almost the same as that when the data for analysis were used. The existence probability of high-growth firms in the four-dimensional area when the data for verification were used was about 0.33 and 0.21 for Table 7 and Table 8, respectively. Although these values are lower than when using the data for analysis, we can assume that high-growth firms are concentrated at a high density, which cannot be considered coincidental. The reason for the lower existence probability of high-growth firms in the four-dimensional area, compared to that for analysis, and the failure of these areas to maintain robustness in the five-dimensional area can be attributed to the fact that the data for verification are one-fifth the number of data for analysis. That is the number of high-growth firms in the area at the four-dimensional area is about 15.7% and 14.0% in Table 7 and Table 8 for validation compared to that for analysis. Thus, the number of high-growth firms in the area is reduced, and the results are no longer stable and robust in high dimensions. The same verification was conducted for the remaining 1034 high-dimensional areas. The distribution of the number of dimensions for which the existence probability of high-growth firms was maximized in the data for analysis and verification was checked (Figure 7).

The numbers in Figure 7 represent the number of areas with each dimension in the analysis and validation data. For example, 77 with a vertical axis of 4 and a horizontal axis of 7 indicates that 77 areas have been extracted in seven-dimensional areas for analysis and verified to four-dimensional areas. Specifically, the area in Table 5 is contained in 72 with a vertical axis of 4 and a horizontal axis of 8, and that in Table 6 is contained in 77 with a vertical axis of 4 and a horizontal axis of 7 in Figure 7. Figure 7 presents that many high-dimensional areas of more than three dimensions are robust for verification. In addition, we can observe a relationship whereby the areas with higher dimensionality for analysis also maintain a higher dimensionality for validation. There was also a 10-dimensional area for which robustness was confirmed up to nine dimensions for verification. The details of this area are provided in Table 9.

The area in Table 9 is the area where the high-growth firms are about 70 times more densely populated than usual for the analysis. This area maintains robustness up to nine dimensions. In the data for verification, the high-growth firms are about 46 times denser than usual in this nine-dimensional area. We also extracted high-dimensional areas that can retain such robustness.

There are 165 areas where the increase in the existence probability of high-growth firms stops at one-dimensional areas for validation, despite that for analysis they are high-dimensional areas with six or more dimensions. In addition, in about half of the 1036 high-dimensional areas, an increase in the existence probability of high-growth firms stopped at three dimensions or less in the data for verification. Therefore, our method exhaustively searches the entire range and extracts local areas.

**Figure 8:**
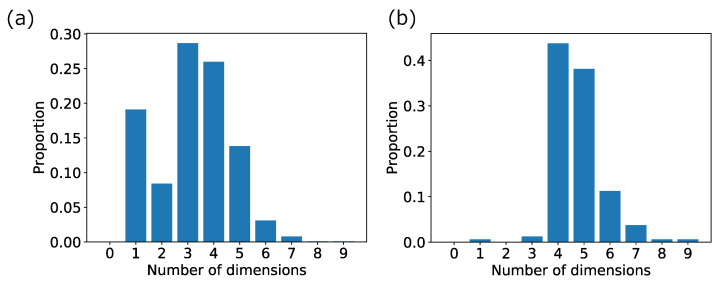
Distribution of the number of dimensions with the maximum existence probability of high-growth firms for verification. The vertical axis and horizontal axes are the proportion of 1036 areas in (**a**) and 160 areas in (**b**) and the number of dimensions, respectively. (**a**) In the 1036 high-dimensional areas. (**b**) In the 160 high-dimensional areas, which include more than 145 high-growth firms.

In the following, we focus on somewhat larger areas wherein the number of high-growth firms includes more than 1% (145 firms) of the total number of high-growth firms in the four-dimensional area in the data for analysis. There were 160 such high-dimensional areas. The areas in Table 5 and Table 9 are included in these 160 areas, but the area in Table 6 is not. The distributions of the number of dimensions with the maximum existence probability of high-growth firms in the 1036 high-dimensional areas and the 160 non-local high-dimensional areas for verification are presented in Figure 8a,b.

As shown in Figure 8, the distribution of the number of dimensions that maximizes the existence probability of high-growth firms in the data for validation has changed significantly by narrowing down from 1036 high-dimensional areas to 160 high-dimensional areas, which include more than 145 high-growth firms. In most of the 160 areas, the number of dimensions in which the existence probability of high-growth firms is maximized in data for verification is four-dimensional or higher. Therefore, in these 160 areas, the robustness can be assumed to be up to four-dimensional. Focusing on these 160 areas, 1–3 in Section 3.2.4 of the method are performed on these areas. The first corresponds to 40 areas, the second to zero areas, and the third to two areas. We finally focused on the 118 four-dimensional areas.

We extracted high-dimensional areas from each of the 29 two-dimensional areas extracted by the five random data. Consequently, we extracted seven three-dimensional areas and 22 two-dimensional areas. The results using the random data are presented in Table 10.

Table 10 shows that we did not extract any high-dimensional areas in any random data. The area with the highest existence probability of high-growth firms among all the random data was the area where high-growth firms were 2.3 times more densely populated than usual. A comparison of the results with the data for analysis indicates that the high-growth firms are much more densely populated than in the random data. Considering that the random data extracted a maximum of only nine areas, the data for analysis, which extracted 1036 high-dimensional areas, showed that the high-growth firms were densely concentrated in many areas. Therefore, we can assume that strong relations exist between high-growing factors of firms and financial items.

### 4.5. Grouping

**Figure 9:**
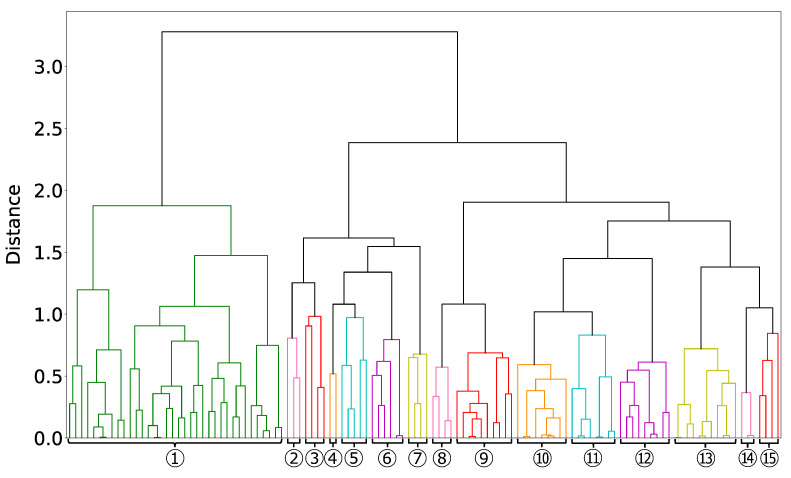
Dendrogram of the result of hierarchical clustering for the 118 four-dimensional areas. The vertical and horizontal axes are the dissimilarity defined using Equation (10) and the result of grouping the 118 four-dimensional areas, respectively. We divided the 118 four-dimensional areas into 15 groups (Groups ① to ⑮). Four-dimensional areas belonging to the same group have a common color. For example, Group ① has green. Groups ② to ⑮ are cyclically painted in six colors.

We define groups of the 118 four-dimensional areas selected in Step4 via hierarchical clustering with the ward method, Step5. The result is presented in Figure 9.

We set the dissimilarity threshold used for grouping in Figure 9 to a value that has a condition common to most of the grouped four-dimensional areas. Thus, the threshold was set to 1, except for the one group on the left, which is grouped because 34 of the 36 four-dimensional areas have the same condition. Finally, we divided the 118 four-dimensional areas into 15 groups. The conditions common to each of the 15 groups are presented in Table 11. We focused on Groups ①, ②, ⑫, and ⑭, which are characteristic among the 15 groups.

Here, 34 of the 36 four-dimensional areas in Group ① have the common condition of small gross profit per capita (less than 2727). The small value indicates that the firms in these 34 areas have small sales and poor operating efficiency. The remaining two four-dimensional areas have the condition that the total capital (compared to all firms in the same industry) is small (smaller than three) and the turnover of total capital (month) is large (larger than 17.79). The total capital (compared to all firms in the same industry) is the value evaluated by TDB and takes the value 0–10. The small value indicates that the total capital is very small compared to other firms in the same industry. The turnover of total capital (month) is the value of total capital divided by sales. Specifically, a large value of that indicates that sales are smaller than the total capital, given that the total capital is very small. Therefore, these two areas extract firms with very small sales and low efficiency. Therefore, the 36 four-dimensional areas in Group ① extract firms with small sales and low operating efficiency. These firms are considered to have improved their operations and increased their sales significantly after three years.

**Figure 10:**
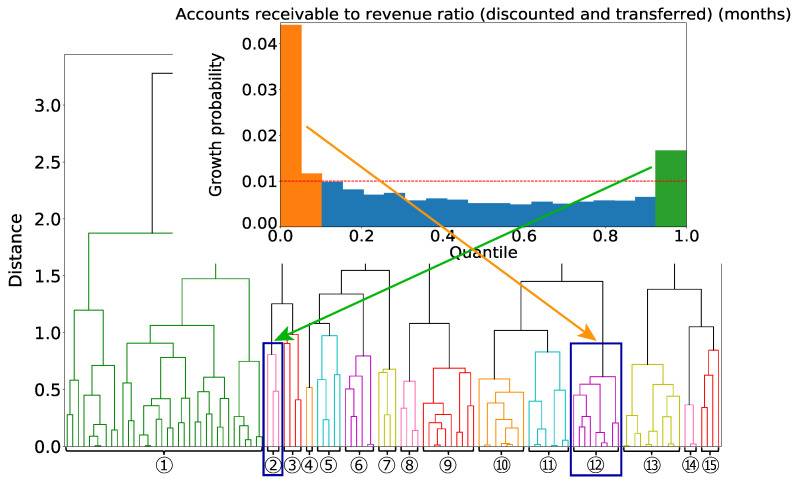
An example of the relation between the groups and a financial item. Group ② is characterized by the green area of the item, the accounts receivable to revenue ratio (discounted and transferred) (months), on the other hand, Group ⑫ is characterized by the orange area.

Next, we focus on Group ② and Group ⑫. These two groups are characterized by different areas of the single variable of the trade receivables (discounted and transferred) turnover periods (months) as shown in Figure 10. Therefore, there is no firm that belongs to both Group ② and Group ⑫.

We consider what type of firms each group is extracting. Group ⑫ has in common the condition that the value of the trade receivables (discounted and transferred) turnover periods (months) is large. This large value implies that the ratio of trade receivables to sales is significant. That is, a firm takes a long time to convert its receivables into cash; thus, firms with insufficient working capital are extracted. In addition, the conditions that the ratio of ordinary income to total assets (industry comparison), turnover of total capital (industry comparison), and ratio of ordinary income to net sales (compared to all firms in the same industry) are bad are extracted together. Thus, we have extracted firms in Group ② that do not have enough working capital and whose profitability is worse. These firms could have improved their operations to afford working capital, which would have led to higher sales. Group ⑫ has in common the condition that the value of the trade receivables (discounted and transferred) turnover periods (months) is small. This small value indicates that, in contrast to Group ②, firms in Group ⑫ can afford working capital. In addition, the conditions that the ratio of ordinary income to total assets (industry comparison) and the ratio of ordinary income to net sales (compared to all firms in the same industry) are bad are extracted together. Therefore, firms in Group ⑫ with low profitability were able to use their surplus working capital to increase sales after three years.

Finally, we focused on Group ⑭. The shared conditions are presented in Table 11. That is, these conditions include the absence of inventories, almost no non-operating income, and very large current assets. In Japan, current assets generally comprise of the following three elements [32]:

- Liquid assets: Short-term fixed deposits, securities, trade notes receivable, trade accounts receivable;
- Inventories: Assets expected to sell on to earn revenue from sales of goods, products, etc.;
- Others: Short-term loans receivable.

Short-term fixed deposits are those with a maturity of one year or less from the closing date. Securities are those with a maturity of one year or less or those held for the short term for trading purposes. Trade notes receivable are promissory notes received as payment for transactions with customers. Trade accounts receivable are accounts receivable from customers for business transactions. Liquid assets are the collective category of these four assets. Inventories are assets that decrease in quantity in the short term that are sold to earn revenue. Short-term loans receivable are loans with a maturity of one year or less from the closing date. Current assets are collectively liquid assets, inventories, and short-term loans receivable. Shared conditions indicate that Group ⑭ firms have large short-term fixed deposits, trade notes receivable, trade accounts receivable, and short-term loans receivable. Therefore, these firms have more assets that can be cashed in within a year. In addition, the conditions of small revenues, small gross profit per employee, and small ordinary income to revenue ratio are extracted together. Hence, we can assume that the firms in Group ⑭ are financially robust and have increased their operating efficiency by making capital investments, developing human resources, and increasing employment, resulting in a significant increase in sales after three years.

## 5. Discussion

We discussed the advantages of using our method. In this study, we first extracted one-dimensional areas, then deleted similar ones, and finally combined the conditions characterizing those areas to extract higher-dimensional crowded data satisfying specific rules. Our method has two advantages. The first is the possibility of extracting combinations of synergistic conditions. In the one-dimensional area extracted in this study, high-growth firms in the most densely populated area were about four times more densely populated than usual, and the average was about 1.7 times more densely populated than usual. However, by combining the conditions, our method can extract areas where the density of high-growth firms is much higher than when the conditions were independent. For example, the two-dimensional area with the highest existence probability of high-growth firms in Table 4 is five times more densely populated with high-growth firms than that when the conditions are independent. Further, the high-dimensional area with the second highest existence probability of high-growth firms in Table 6 is seven times more densely populated with high-growth firms than that when all conditions are independent. Thus, our method can exhaustively extract combinations that seem to have synergistic effects.

Second, our method can also extract local areas and robust high-dimensional ones. In this study, we focused on somewhat larger areas to analyze universal factors, but we also extracted local areas. For example, we extracted the areas on the left side in Figure 6 where the existence probability of high-growth firms is lower than other extracted high-dimensional areas. We also extracted the high-dimensional areas at the bottom in Figure 7 that can only validate up to low dimensions due to insufficient data for verification. Contrary to this study, we can use our method if we want to focus on local and specific cases, rather than universal ones. In addition, we can extract localized areas and areas with robustness. For example, we extracted the high-dimensional area with strong robustness (Table 9). We can use our method when we want to focus on something universal, as in this study.

We discussed some of the considerations for this study. After the extraction of high-dimensional areas, we selected four dimensions as the number of dimensions that could withstand verification. First, we discussed regarding the extraction of high-dimension areas. Meanwhile, we extracted the areas of seven or more dimensions, in the data for verification, more than half of all extracted areas where the increase in the existence probability of high-growth firms stopped at three dimensions (see Figure 8a). There are two reasons for this. The first one is that there were cases where the number of firms was small in the initially extracted areas because our method performed an exhaustive search that includes local areas. The second one is that the increase in the existence probability of high-growth firms tends to stop since the data for verification is one-fifth of the data for analysis in terms of the number of data. Therefore, if it is not a local area, we can increase the number of dimensions that allow verification by increasing the data for verification to about the same number as that for analysis. Second, we discussed the number of dimensions that we used. While increasing the number of dimensions that allow verification by increasing the data for verification, considering that the area tends to be localized is necessary. In this study, to focus on areas where firms universally tend to high growth, we focused on 160 four-dimensional ones where more than 1% of the total number of high-growth firms existed. Considering that we initially extracted 1036 high-dimensional areas, clearly that our method can easily extract localized areas. Therefore, determining to what dimensionality the results should be validated and used as universal results is necessary.

**Figure 11:**
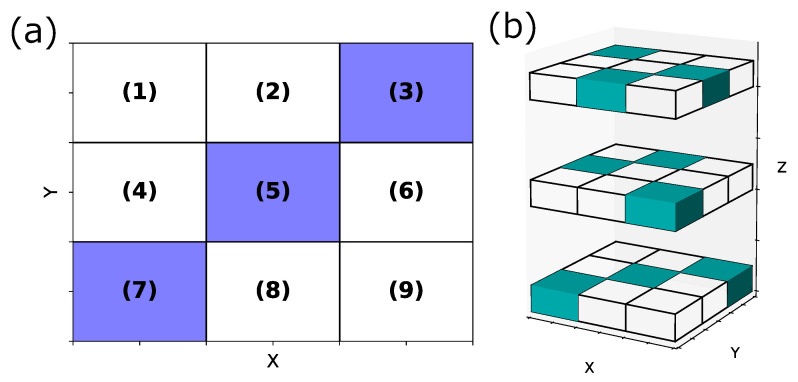
Examples of missing dense areas with this method. The colored areas are where data that satisfy specific conditions are densely distributed. (**a**) Example of missing in two dimensions. (**b**) Example of missing in three dimensions.

We also discussed some concerns when using our method. In this study, we first extracted one-dimensional areas, then deleted similar ones, and finally combined the conditions characterizing those areas to extract higher-dimensional crowded with data satisfying specific rules. However, if the densification occurs in the way shown in the following Figure 11a,b, we miss dense areas.

In Figure 11a,b, we divided each axis into three parts. Data satisfying specific conditions were densely populated in the colored areas in these figures. In Figure 11a, the case of the missing dense area is when the existence probability of data satisfying specific conditions in areas (1)∼(3), (4)∼(6), and (7)∼(9) is equal. In this case, when projected onto the Y-axis, we cannot extract the area on the Y-axis. Thus, we cannot extract the two-dimensional areas (3), (5), and (7). We also consider the case where (1)∼(3) > (4)∼(6) > (7)∼(9) in terms of the density of data satisfying specific conditions between areas (1)∼(3), (4)∼(6) and (7) ∼(9). We consider areas (7)∼(9) as the areas where data satisfying the specified conditions are not dense on the Y-axis, and we cannot extract area (7). The possibility exists that a similar phenomenon may occur in the third dimension and beyond. In the case of Figure 11b, as in the previous case, if the existence probability of data satisfying specific conditions is equal in the three divisions in any of the X-, Y-, and Z-axis directions, we cannot extract the colored areas in Figure 11b.

We can consider a possible method to address this concern to start focusing on two or higher dimensions, rather than focusing on one dimension. In a pair that selects two from all variables, we can address this by dividing the area, calculating the existence probability of data satisfying specific conditions in each area, and extracting the areas with a higher density of data satisfying certain conditions than normal ones. In Figure 11a, we can extract areas (3), (5), and (7) by calculating the existence probability of data satisfying specific conditions in each of areas (1)∼(9). In Figure 11b, we can extract the colored areas by calculating the existence probability of data satisfying specific conditions in each of the 27 areas. Meanwhile, since this method requires considering all variable partitions and calculating the probability in each of them, we predicted a significant increase in computational cost. Specifically, we considered the case where we divide each financial item by 5% as in this study and searched in two dimensions, as shown in Figure 11a, to avoid missing anything in dense areas. In this case, we divided each financial item by a maximum of 20 and considered the 12,090 combinations of selecting two from all 156 financial items. Therefore, it is necessary to calculate the existence probability of data satisfying specific conditions in a maximum of 400 areas in each combination, totaling a maximum of about 4.8 million areas. We also considered the case of focusing on three dimensions, as shown in Figure 11b. We considered the 620,620 combinations of selecting three from all 156 financial items. Therefore, it is necessary to calculate the existence probability of data satisfying specific conditions in a maximum of 8000 areas in each combination, totaling a maximum of about 3 billion areas. Thus, the computational cost increases exponentially as we increase the number of dimensions that we begin to focus on. Therefore, we consider this method of addressing this problem when only a few variables exist. However, even if we searched exhaustively for a specific dimension, the same problem can occur above that dimension and beyond. Specifically, Figure 11b shows an example where a miss occurs in some three-dimensional areas, regardless of whether one starts looking at a one-dimensional or two-dimensional area. Therefore, we must discuss which dimension to examine exhaustively and which dimension and beyond to ignore invisible relationships.

We compared some popular existing methods with our method for comparison. In high-dimensional areas, when data satisfying specific conditions are concentrated in multiple areas, we call the problem of extracting all areas the multimodality problem. In the special case that there is only one highly concentrated area in the whole space, we call it a unimodality problem. For unimodality problems, we can extract the dense area by using popular methods such as multiple regression analysis or support vector machines. However, these methods are not suitable for the analysis of high-growth firms in this study, as we showed in Section 4, there are at least 15 dense areas in the 156-dimensional space. In addition, other popular methods, neural networks [33], are black-box methods, making it impossible to interpret the results in terms of important financial items. Random forests are also popular in big data analysis; however, they are unsuitable for the present problem of extracting important factors in the form of sets of variables. Our method can extract the sets of important factors for multimodality problems and is suitable for the analysis of high-growth firms.

We also compared the factors extracted in this study to Coad’s previous study [28]. In that study, they used cluster analysis, which is strong for multimodality problems, to analyze the important factors of high-growth firms. Although the high-growth firms in the previous study are about 2% of the total data, we note that the definitions of high-growth firms and the variables used are very different. The previous study found that firms with low inventories, higher previous employment growth, and higher short-term liabilities are more likely considered high-growth firms. As previous employment growth is excluded from the financial item of this study, we analyzed other results. We identified the factor of low inventory as a universal factor in Group ⑩ and Group ⑭ of this study (see Figure 9 and Table 11). We extracted the factor of higher short-term liabilities in the high-dimensional area of Table 9. Therefore, we can assume that we have extracted the same results as in the previous studies.

We also compared the factors extracted in this study to that of Delemar’s previous study [26]. In that study, they used Lasso, which is strong for unimodality problems, to analyze the important factors involved in forecasting high-growth firms. We note that the definition of high-growth firms differs from the previous study and the variables used are also very different. After comparing the results with this assumption, we extracted similar results to the previous study for increasing employment. In the previous study, increasing employment was part of the factors for the seven clusters of high-growth firms. The firms in Group ⑭ in this study are financially robust and have increased their operating efficiency by making capital investments, developing human resources, and increasing employment. Therefore, we believe that the result extracted in this study is similar to the previous one. The previous study focused on revenue growth. However, in this study, we extracted the areas that focused on this as localized areas, with the number of high-growth firms being less than 100 in any two-dimensional ones. The study was different from previous studies that extracted revenue growth as universal.

Finally, we analyzed the indicators used in our method. For the 15 groups extracted using our method, we found the poor operating efficiency for most groups. The possible reason is that we used the top 1% of all firms in sales growth rate as the definition of high-growth firms. Firms with approximately four times or higher sales after three years often have either a pattern; that is, firms with poor operating efficiency have succeeded in improving their sales or sales are small from the start. Thus, we may need to change the definition of high-growth firms. In addition, we measured firm growth in this study using the absolute one in sales over three years. As sales are not a perfect indicator [26], some studies used the number of employees [21,34] and both the number of employees and sales [35]. Therefore, discussing which items we should use as a measure of growth and what should be the definition of a high-growing firm is necessary.

## 6. Conclusions

We introduced a new non-black-box method of extracting multiple areas in a high-dimensional big data space where data points that satisfy specific conditions are highly concentrated. We analyzed high-growth firms in all industries as an example of the applications in this study. We categorized the high-growth firms into 15 groups of different sets of factors. Conducting factor analysis of high-growth firms in specific industries or firms that have gone bankrupt by using this method is feasible. In addition, this method is not limited to corporate data and can be applied to various fields of analysis, including the use of medical data for predicting diseases based on genetic changes.

## Figures and Tables

**Table 1:** Abbreviated names of items, units, and indicators.

Abbreviated Name	Item Name
OIR	Ordinary income to revenue ratio
CLR	Current liabilities to revenue ratio
OITC	Ordinary income to total capital ratio
LR	Liabilities to revenue ratio
NIR	Net income to revenue ratio
CACL	Current assets to current liabilities ratio
LACL	Liquid assets to current liabilities ratio
CGSR	Cost of goods sold to revenue ratio
GPE	Gross profit per employee
TCR	Total capital to revenue ratio
FAR	Fixed assets to revenue ratio
FAFL	Fixed assets to fixed liability ratio
NOLR	Non-operating loss to revenue ratio
IR	Inventories to revenue ratio
CAR	Current assets to revenue ratio
APR	Accounts payable to revenue ratio
ARR	Accounts receivable to revenue ratio
PPER	Property, plant and equipment to revenue ratio
NCLR	Not current liabilities to revenue ratio
DR	Depreciation to revenue ratio
CFS	Compared to all firms in the same industry
IC	Industry comparison
DT	After discounting and transferring
DA	In data for analysis
DV	In data for verification
NAE-nD	Number of areas extracted in n-D
NDEHA	Number of dimensions of each high-dimensional areas
NC	Number of conditions
NF	Number of firms
NHF	Number of high-growth firms
EPHF	Existence probability of high-growth firms
M	Months
T	Thousands of yen

**Table 2:** Top five areas in the 197 areas of 143 financial items extracted in Step1. The extracted areas are from lower to upper limits. The lower and upper limits are denoted by percentage points within the financial item. The existence probability of high-growth firms (EPHF) in an area is calculated using the number of high-growth firms in the area, the number of all firms in the area, and Equation (5). The abbreviated names used in this table are defined in Table 1.

Item Name	Lower Limit	Upper Limit	NHF	NF	EPHF
OIR (CFS)	0.0%	6.7%	4219	96,108	0.042
OIR (IC)	0.0%	6.7%	4206	96,248	0.042
CLR (M)	91.9%	100.0%	4309	117,124	0.036
OITC (IC)	0.0%	6.6%	3509	95,920	0.035
LR (M)	90.8%	100.0%	4650	132,567	0.035

**Table 3:** Two areas extracted in the ratio of net income to sales (before amortization and after tax, %), orange and green, respectively, in Figure 3. The extracted areas are from lower to upper limits, which are denoted by percentage points within the financial item. The existence probability of high-growth firms of an area is calculated using the number of high-growth firms in the area, the number of all firms in the area, and Equation (5). The abbreviated names used in this table are defined in Table 1.

Area	Lower Limit	Upper Limit	NHF	NF	EPHF
orange	0.0%	28.9%	7203	417805	0.017
green	94.6%	100.0%	1439	77645	0.017

**Table 4:** Top five two-dimensional areas in Step3. The existence probability of high-growth firms of an area is calculated using the number of high-growth firms in the area, the number of all firms in the area, and Equation (5). The ratio in this table is the existence probability of high-growth firms in two dimensions divided by the existence probability of high-growth firms calculated given that the two conditions are independent using Equation (8). The abbreviated names used in this table are defined in Table 1.

Item Name	EPHF(1D)	Item Name	EPHF(1D)	EPHF(2D)	Ratio
CLR (M)	0.036	CACL (%)	0.013	0.199	5.142
CLR (M)	0.036	LACL (%)	0.012	0.196	5.232
CSGR (%)	0.025	GPE (T)	0.020	0.177	5.147
TCR (M)	0.022	FAR (M)	0.019	0.174	5.647
FAFL (%)	0.024	FAR (M)	0.023	0.173	4.702

**Table 5:** Eight-dimensional area with the first highest existence probability of high-growth firms among the extracted high-dimensional areas. The ratio in this table is the existence probability of high-growth firms in the *n*-dimensional area divided by that of high-growth firms calculated under conditions where the *n*-conditions are independent using Equation (8); *n* is the number of conditions in the row (Column NC). The abbreviated names used in this table are defined in Table 1.

NC	Item Name (Threshold)	NHF	EPHF	Ratio
1	NOLR (%) (≤0)	3715	0.031	1.000
2	IR (M) (≤0)	2447	0.066	1.276
3	CAR (M) (≥10.2)	664	0.161	2.182
4	OITC (IC) (≤2)	217	0.408	4.194
5	GPE (T) (≤2727)	112	0.530	4.984
6	FAR (M) (≥10.19)	40	0.673	5.699
7	APR (M) (≤0)	33	0.748	5.382
8	CLR (M) (≥7.44)	26	0.779	4.837
9	OIR (CFS) (≤2)	26	0.779	4.133

**Table 6:** Seven-dimensional area with the second highest existence probability of high-growth firms among the extracted high-dimensional areas. The ratio in this table is the existence probability of high-growth firms in the *n*-dimensional area divided by that of high-growth firms calculated under conditions where the *n*-conditions are independent using Equation (8); *n* is the number of conditions in the row (Column NC). The abbreviated names used in this table are defined in Table 1.

NC	Item Name (Threshold)	NHF	EPHF	Ratio
1	ARR (DT) (M) (≤0.25)	4336	0.028	1.000
2	Revenue to total capital ratio (IC) (≤3)	1790	0.058	1.604
3	OITC (IC) (≤2)	550	0.215	3.548
4	PPER (M) (≤0.16)	136	0.475	6.658
5	NCLR (M) (≤0)	92	0.606	7.117
6	Investment and financing returns (%) (≤0.02)	60	0.683	7.272
7	DR (%) (≤0)	37	0.771	7.322
8	OIR (CFS) (≤2)	36	0.765	5.690

**Table 7:** Validation result for the high-dimensional area of Table 5 with the highest existence probability of high-growth firms. We add conditions in the same order as in Table 5 until the existence probability of high-growth firms stops to increase. The abbreviated names used in this table are defined in Table 1.

NC	Item Name	Threshold	NHF	EPHF
1	NOLR (%)	≤0	551	0.031
2	IR (M)	≤0	379	0.064
3	CAR (M)	≥0.122	106	0.074
4	OITC (IC)	≤2	34	0.334
5	GPE (T)	≤2727	10	0.204

**Table 8:** Validation result for the high-dimensional area of Table 6 with the second-highest existence probability of high-growth firms. We add conditions in the same order as in Table 6 until the existence probability of high-growth firms stops to increase. The abbreviated names used in this table are defined in Table 1.

NC	Item Name	Threshold	NHF	EPHF
1	ARR (DT) (M)	≤0.25	680	0.029
2	Revenue to total capital ratio (IC)	≤3	233	0.046
3	OITC (IC)	≤2	79	0.183
4	PPER (M)	≤0.16	19	0.208
5	NCLR (M)	≤0	11	0.207

**Table 9:** Ten-dimensional area for which robustness was confirmed in up to nine dimensions for verification. The abbreviated names used in this table are defined in Table 1.

NC	Item Name (Threshold)	NHF (DA)	EPHF (DA)	NHF (DV)	EPHF (DV)
1	Revenue (T) (≤108,917)	9577	0.032	1253	0.041
2	NOLR (%) (≤0)	3156	0.056	450	0.065
3	CAR (M) (≥10.2)	1046	0.134	143	0.123
4	OITC (IC) (≤2)	338	0.278	51	0.234
5	PPER (M) (≤0.16)	137	0.471	25	0.317
6	LR (M) (≥14.13)	78	0.562	17	0.358
7	NCLR (M) (≤0)	63	0.672	11	0.371
8	Revenue to total capital ratio (IC) (≤ 3)	59	0.691	11	0.404
9	IR (M) (≤0)	37	0.694	10	0.464
10	LACL (%)(≤41.45)	18	0.700	3	0.144
11	OIR (CFS) (≤2)	18	0.700		

**Table 10:** Results using random data. EPHF represents the value of the existence probability of high-growth firms in the area where the existence probability of high-growth firms is the highest among the extracted high-dimensional areas.

Data	NAE-1D	NAE-2D	NDEHA	EPHF
1	11	3	2, 2, 3	0.0140
2	11	7	2, 2, 2, 2, 2, 2, 3	0.0226
3	12	9	2, 2, 2, 2, 2, 2, 2, 3, 3	0.0161
4	13	4	2, 2, 2, 2	0.0167
5	13	6	2, 2, 2, 3, 3, 3	0.0152

**Table 11:** Conditions common to each of the 15 groups. The abbreviated names used in this table are defined in Table 1. If the variables common to a group include those with an alphabet in front of the variable name, all four-dimensional areas in the group have in common that one or more of them are satisfied. For example, all four-dimensional areas in Group ⑤ contain the condition of the turnover of current assets and one or more of either (a) or (b). If the variables common to a group include variables with an alphabet with tilde in front of the variable name, all four-dimensional areas in the group have in common that two or more conditions are satisfied in them. For example, all four-dimensional areas in Group ⑮ contain two or more of the conditions ã, $\tilde{b}$, or $\tilde{c}$.

Group	Item name	Threshold
①	GPE (T)	≤2727
②	ARR (DT) (M)	≥3.86
③	PPER (M) (a) Financial account to revenue ratio (%) (b) DR (%)	≤0.00 ≤0.00 ≤0.00
④	Cash and deposits to revenue ratio (days) OITC (IC)	≥130.33 ≤2.00
⑤	CAR (M) (a) Interest coverage ratio (times) (b) Capital to revenue ratio (M)	≥10.20 ≤−8.49 ≤−0.81
⑥	OITC (IC) (a) CACL (%) (b) NCLR (M) (c) Capital to revenue ratio (M)	≤2.00 ≤78.68 ≤0.00 ≤−0.81
⑦	CAR (M) Non-operating income to revenue ratio (%)	≥10.20 ≥4.62
⑧	CAR (M) DR (%)	≥10.20 ≤0.00
⑨	PPER (M) (a) TCR (M) (b) Revenue to total capital ratio (IC) (c) OIR (CFS)	≤0.16 ≥17.79 ≤3.00 ≤2.00
⑩	IR (M)	≤0.00
⑪	CAR (M) ($\tilde{a}$) Non-operating income to revenue ratio (%) ($\tilde{b}$) OITC (IC) ($\tilde{c}$) APR (M)	≥10.2 ≤0.00 ≤2.00 ≤0.00
⑫	ARR (DT) (M)	≤0.25
⑬	CAR (M) (a) Financial account to revenue ratio (%) (b) Investment and financing returns (%)	≥10.20 ≤0.03 ≤0.02
⑭	CAR (M) IR (M) Non-operating income to revenue ratio (%)	≥10.20 ≤0.00 ≤0.05
⑮	($\tilde{a}$) Total capital (CFS) ($\tilde{b}$) Investment and financing returns (%) ($\tilde{c}$) Capital to revenue ratio (M)	≤3 ≤0.02 ≥8.53

## Data Availability

Restrictions apply to the availability of these data. The data were obtained from TEIKOKU DATABANK, Ltd. (chuo-ku, Tokyo 104-8685 ) and are available from the authors with the permission of TEIKOKU DATABANK, Ltd.
